# Massive Ovarian Edema in a Girl with Hemoglobin SC Disease

**DOI:** 10.1155/2018/4193248

**Published:** 2018-03-14

**Authors:** Eric Johannesen, Van Nguyen

**Affiliations:** Department of Pathology and Anatomical Sciences, University of Missouri, Columbia, MO 65203, USA

## Abstract

Massive ovarian edema is a benign tumor like lesion of the ovary. The widely accepted mechanism is disruption of vascular drainage resulting in accumulation of fluid within the stroma and enlargement of the ovary. We report a case of massive ovarian edema in a teenage girl with hemoglobin SC disease. A 16-year-old female with hemoglobin SC disease was admitted with right lower quadrant pain. An ultrasound and CT scan showed a large, heterogeneous solid, and cystic pelvic mass. Due to the size and the possibility of malignancy, the patient underwent a salpingo-oophorectomy. The mass was an 8.3 cm hemorrhagic cyst with some solid areas. Histologic exam showed diffuse edema with scattered entrapped follicles and a narrow rim of normal appearing ovarian stroma. Dilated and occluded capillaries were seen along with hemorrhage and sickled red blood cells but no necrosis was identified. These histologic features were consistent with massive ovarian edema. Massive ovarian edema is thought to be caused by disturbance of the vascular outflow resulting in fluid buildup in the stroma. It is most often attributed to intermittent ovarian torsion that disrupts capillary and venous flow, but arterial flow is maintained. Rare cases of massive ovarian edema caused by tumor emboli or external compression by tumors have been reported, but this is the first case of a patient with hemoglobin SC disease developing vasoocclusions resulting in this lesion.

## 1. Introduction

Massive ovarian edema is benign tumor like lesion of the ovary caused by the accumulation of interstitial fluid resulting in ovarian enlargement. It is widely thought to be caused by the obstruction of lymphatics and veins, which results in the leakage of fluid out of the vessels. It is most often caused by external compression by neoplastic or polycystic ovaries with intravascular obstruction being less common. This is a unique case of massive ovarian edema caused by a vasoocclusive crisis in a girl with hemoglobin SC disease.

## 2. Clinical Case

A 16-year-old female was admitted to the hospital with a complaint of acute abdominal pain and vomiting. Her past medical history is significant for hemoglobin SC disease. She has had episodes of acute chest syndrome with the most recent in the fall of 2013. Her abdominal pain was described as sharp and was localized to the right lower quadrant with radiation on her right lower back. She states that although she has had abdominal pain caused by sickle cell crisis before, this current pain feels different. An ultrasound and CT scan was performed which showed a large, heterogeneous solid, and cystic pelvic mass. The differential diagnosis ranged from a teratoma to a germ cell tumor. Due to the size of the mass and the concern for malignancy, the patient underwent a salpingo-oophorectomy.

## 3. Operative Findings

A large, freely mobile dark purple mass with a translucent wall was seen originating from the right ovary. Upon attempt to remove the mass, a cyst ruptured, spilling straw colored fluid. After removal from the abdomen, the cyst was also found to contain clotted blood. No other abnormalities were seen within the abdomen or pelvis.

## 4. Pathologic Findings

**Figure 1:**
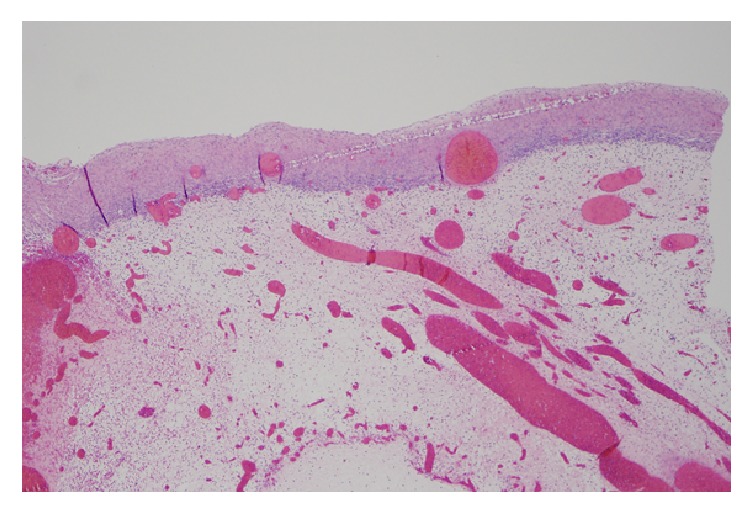
Medium power view showing edema with rim of ovarian stroma with stromal luteinization.

**Figure 2:**
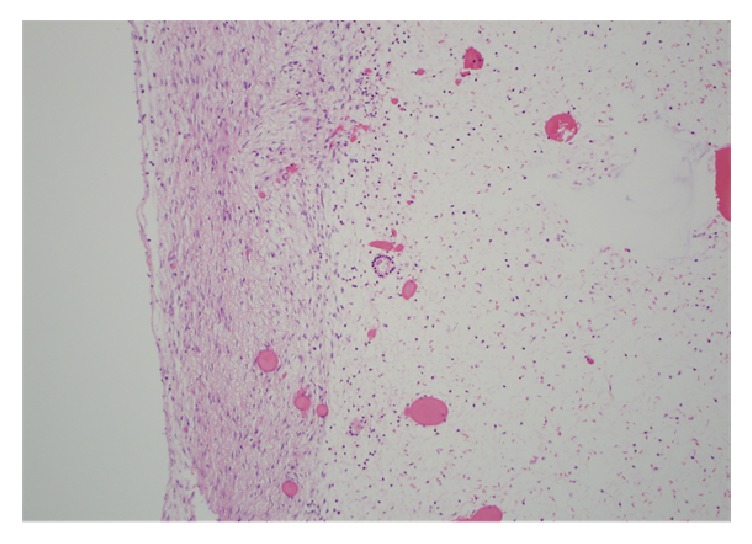
Medium power view showing an ovarian follicle surrounded by edema.

**Figure 3:**
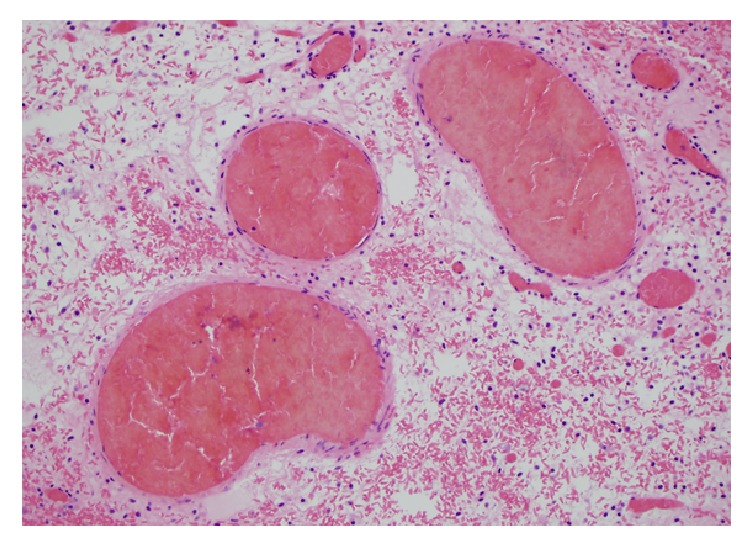
Medium power view of thrombosed vessels with extensive interstitial hemorrhage.

**Figure 4:**
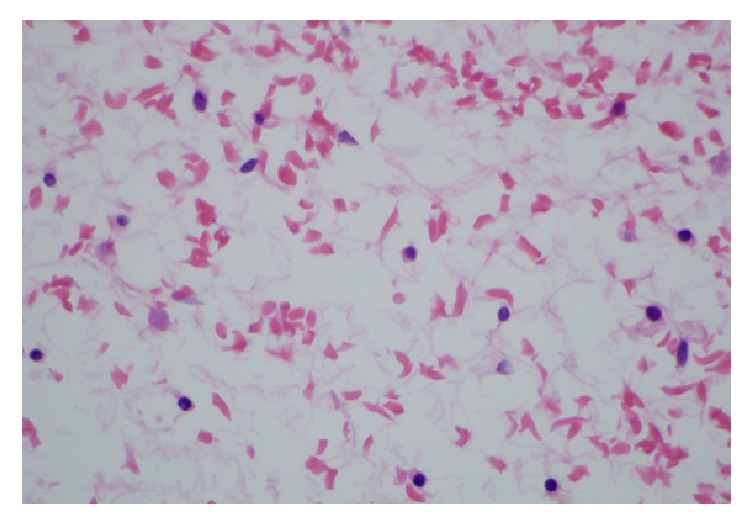
High power view showing sickled and tetragonal erythrocytes.

Grossly, the mass was an 8.3 cm in greatest dimension diffusely hemorrhagic cyst with some solid areas. Cut surfaces showed diffusely hemorrhagic tissue with no recognizable ovarian tissue seen. Histologic exam showed extensive edema throughout the ovary. A narrow rim of ovarian stroma with luteinization was present along the outer surface (Figure 1). Rare follicles were seen within the edematous areas (Figure 2). Extensive vascular ectasia with numerous thrombosed capillaries was seen (Figure 3) along with numerous sickled and tetragonal erythrocytes (Figure 4). No evidence of any neoplasms or other primary ovarian pathology was seen. The overall histologic features were consistent with massive ovarian edema caused by vasoocclusive crisis due to her hemoglobin SC disease.

## 5. Discussion

Massive ovarian edema is defined as the extreme enlargement of the ovary due to the accumulation of fluid within the interstitial space. The ovary has an extensive vascular network, most likely due to the fact that it is both a recipient and producer of hormones. If drainage within the lymphatic and/or venous component is disrupted then fluid will move out into the interstitial tissue [1]. Since arterial flow is maintained this process is usually asymptomatic. However, if the disruption is not corrected, the ovary will continue to enlarge and either compress on adjacent structures, mimicking a neoplastic process, or undergo torsion resulting in disruption of the ovary's arterial supply. If the torsion occurs and is not quickly corrected then infarction will result. Massive ovarian edema has been divided into primary and secondary types based on the original condition of the affected ovary [2]. Primary type is massive edema in an otherwise normal ovary. Examples of primary types that have been reported in the literature include a leiomyoma within the broad ligament, acute appendicitis, and vascular occlusion by metastatic cancer cells [3–5]. In the secondary type, the ovary has a preexisting pathologic process that causes or contributes to the edema [2]. Examples of the secondary type are primary ovarian neoplasms like cystadenomas and non-neoplastic tumor like lesions such as polycystic ovaries [6, 7]. The classic histologic features of massive edema are diffuse edema that surrounds, not displaces, normal ovarian structures and a narrow rim of uninvolved ovarian stroma seen just under the outer surface. Another feature frequently seen is stromal luteinization, a phenomenon that is hypothesized to be the result of tension placed on the cells by the intercellular fluid [3]. In the current case, the luteinized cells were in the subcapsular stroma, not the edematous areas, which appears to contradict this hypothesis.

Our case is unique in that it was caused by complications of hemoglobin SC disease, a condition that to our knowledge has not been previously reported. Hemoglobin SC disease is the second most common form of sickle cell disease, comprising about 30% of cases [8]. It is defined as the presence of hemoglobin S and hemoglobin B in equal concentrations within the erythrocyte. Red blood cells that contain hemoglobin C tend to be dehydrated because of constant loss of potassium and water due to upregulation of K-Cl channels. The loss of water causes the relative concentration of hemoglobin S within the cell to increase, which leads to polymerization of the hemoglobin and red cell deformation [9, 10]. The clinical complications of sickle cell disease are centered on the damage to organs due to vascular occlusion. The reason for this appears to be that hemoglobin SC disease tends to promote inflammation and coagulation. Studies have shown that persons with hemoglobin SC disease tend to have higher expression of tissue factor on circulating leukocytes. They also tend to have increased levels of thrombomodulin, VCAM-1, and TNF-alpha, which are involved in upregulating adhesive molecules on platelets and endothelial cells [11, 12]. Extracellular hemoglobin released by hemolysis also causes increased consumption of nitric oxide and stimulates circulating neutrophils to release uncondensed chromatin and enzymes, which further exacerbates the hypercoagulable state [13, 14]. Clinically, patients with hemoglobin SC disease are susceptible to the same complications as hemoglobin SS patients, but the affected sites, onset, and frequency differ. Hemoglobin SC patients tend to have less severe hemolysis; therefore, complications related to hemolysis, such as cholecystitis, are less common. However, complications related to hypercoagulation, such as osteonecrosis, tend to be as frequent or, in instances of acute chest syndrome, may be even more frequent than hemoglobin SS [8, 9].

The hypercoagulable conditions associated with hemoglobin SC disease along with the histologic features seen in the patient's ovary give clues to the pathogenesis.

In this patient, the process most likely started within the small veins and lymphatics. The erythrocytes and neutrophils with their hypercoagulable properties began to adhere to the endothelium within these vessels. Eventually, these vessels became occluded, which increased the hydrostatic pressure resulting in fluid leaking out into the ovarian stroma. The disruption of the vascular drainage in the affected areas most likely caused release of inflammatory mediators, which then resulted in more vascular obstruction and more edema. The swelling then progressed until the ovary enlarged to the extent that it began to undergo torsion, which mechanically compromised arterial flow. This resulted in the symptomatic abdominal pain felt by the patient, which necessitated her presentation to the emergency room.

Massive ovarian edema is enlargement of the ovary due to excessive interstitial fluid. It is caused by either intravascular obstruction or mechanical compression of lymphatics and small veins resulting in fluid extravasation. Hemoglobin SC disease is the second most common form of sickle cell disease. It is associated with hypercoagulative states, which can lead to vasoocclusive crises. This case is an example of a patient with hemoglobin SC disease developing primary massive ovarian edema caused by venous and capillary thrombi in a background of inflammation and hypercoagulation.

## References

[1] Brown H. M., Russell D. L. (2014). Blood and lymphatic vasculature in the ovary: Development, function and disease. *Human Reproduction Update*.

[2] Praveen R. S., Pallavi V. R., Rajashekar K. (2013). A clinical update on massive ovarian oedema a pseudotumour?. *Ecancer*.

[3] Harrison B. T., Berg R. E., Mittal K. (2014). Massive ovarian edema associated with a broad ligament leiomyoma: A case report and review. *International Journal of Gynecological Pathology*.

[4] Callen A. L., Illangasekare T., Poder L. (2017). Massive ovarian edema, due to adjacent appendicitis. *Emergency Radiology*.

[5] Bazot M., Detchev R., Cortez A., Uzan S., Darai E. (2003). Massive ovarian edema revealing gastric carcinoma: A case report. *Gynecologic Oncology*.

[6] Thomas R. L., Carr B. R., Ziadie M. S., Wilson E. E. (2012). Bilateral mucinous cystadenomas and massive edema of the ovaries in a virilized adolescent girl.. *Obstetrics & Gynecology*.

[7] Guvenal T., Cetin A., Tasyurt A. (2001). Unilateral massive ovarian edema and polycystic ovaries. *European Journal of Obstetrics & Gynecology and Reproductive Biology*.

[8] Rees D. C., Williams T. N., Gladwin M. T. (2010). Sickle-cell disease. *The Lancet*.

[9] Nagel R. L., Fabry M. E., Steinberg M. H. (2003). The paradox of hemoglobin SC disease. *Blood Reviews*.

[10] Hannemann A., Weiss E., Rees D. C., Dalibalta S., Ellory J. C., Gibson J. S. (2011). The properties of red blood cells from patients heterozygous for HbS and HbC (HbSC Genotype). *Anemia*.

[11] Colella M. P., de Paula E. V., Machado-Neto J. A. (2015). Elevated hypercoagulability markers in hemoglobin SC disease. *Haematologica*.

[12] Zhang D., Xu C., Manwani D., Frenette P. S. (2016). Neutrophils, platelets, and inflammatory pathways at the nexus of sickle cell disease pathophysiology. *Blood*.

[13] Zhou Z., Behymer M., Guchhait P. (2011). Role of extracellular hemoglobin in thrombosis and vascular occlusion in patients with sickle cell anemia. *Anemia*.

[14] Chen G., Zhang D., Fuchs T. A., Manwani D., Wagner D. D., Frenette P. S. (2014). Heme-induced neutrophil extracellular traps contribute to the pathogenesis of sickle cell disease. *Blood*.

